# Development and preliminary tests of a portable volumetric capnograph for outpatient use

**DOI:** 10.36416/1806-3756/e20250136

**Published:** 2025-11-19

**Authors:** Francisco Ubaldo Vieira, Denilson Antônio Marques, Natalie Camila dos Reis Silva, Maria Ângela Gonçalves de Oliveira Ribeiro, Marcos Melo Moreira, Ilma Aparecida Paschoal, Isadora Minuzzi Vieira, Eduardo Tavares Costa

**Affiliations:** 1. Instituto Federal de Educação, Ciência e Tecnologia de São Paulo - IFSP - Campus Campinas, Campinas (SP) Brasil.; 2. Centro de Engenharia Biomédica - CEB - Universidade Estadual de Campinas - UNICAMP - Campinas (SP) Brasil.; 3. Faculdade de Engenharia Elétrica e de Computação, Universidade Estadual de Campinas - UNICAMP - Campinas (SP) Brasil.; 4. Faculdade de Ciências Médicas, Universidade Estadual de Campinas - UNICAMP - Campinas (SP) Brasil.

**Keywords:** Volumetric capnography, microcontroller, respiratory monitoring, spontaneous breathing, portable device

## Abstract

**Objective:**

To develop and validate a portable volumetric capnograph for collecting data on ventilatory mechanics during spontaneous breathing for outpatient use.

**Methods:**

The device was developed by integrating the following commercially available sensors: a Hamilton^®^ flow sensor (variable orifice; Hamilton Medical AG, Graubünden, Switzerland); an SDP810-125PA differential pressure sensor (Sensirion AG, Stäfa, Switzerland); and a Capnostat 5 CO_2_ sensor (Philips Respironics, Murrysville, PA, USA). An Arduino UNO-R3^®^ microcontroller (Arduino, Monza, Italy) was used as an interface between the sensors and a laptop computer, and a Python application was used to acquire data at 10 ms intervals (100 Hz). Validation included static tests (flow: 0-45 L/min; partial pressure of CO_2_: 0-100 mmHg) and tests with five healthy volunteers (n = 115 respiratory cycles), in comparison with the reference equipment (a CO_2_SMO Plus® DX-8100 oxycapnograph; Philips Respironics).

**Results:**

The static tests showed excellent linear correlation for flow and CO_2_ concentration. For the tests conducted with the five volunteers, no significant differences were observed between the portable volumetric capnograph and the reference equipment for any of the variables analyzed. Intracycle variability was observed in the capnography curves, reflecting the physiological characteristics of spontaneous breathing.

**Conclusions:**

Our portable volumetric capnograph demonstrated the ability to collect accurate data on flow and partial pressure of CO_2_ during spontaneous breathing, with performance equivalent to that of the reference equipment. The variability in the capnography curves represents an intrinsic characteristic of spontaneous breathing that must be considered when developing algorithms for calculating physiological indicators.

## INTRODUCTION

Chronic respiratory diseases are increasing significantly worldwide and represent a global public health challenge, affecting millions of people annually.^1^ ^,^ ^2^ Among the main conditions are asthma and COPD, which impose a significant socioeconomic burden worldwide.^3^ In the United States, approximately 14.2 million adults were diagnosed with COPD in 2021, highlighting the magnitude of this problem.^4^

Spirometry has traditionally been considered the gold standard for assessing lung function.^5^ ^,^ ^6^ However, this method has important limitations, including dependence on the ability of patients to perform forced expiratory maneuvers^7^ and a shortage of qualified professionals to perform it.^8^ ^,^ ^9^ Spirometry requires maximum expiratory effort, which can be difficult or impossible for patients with severe dyspnea, chest pain, or cognitive limitations.^10^

Volumetric capnography is emerging as a promising alternative for the diagnosis and monitoring of chronic lung diseases.^11^ ^-^ ^14^ While temporal capnography measures CO_2_ concentration over time, volumetric capnography incorporates information on expired volume, providing more comprehensive data on respiratory function.^15^ ^,^ ^16^ This technique offers significant advantages, including the fact that it is noninvasive, has low cost, and requires only 10 ventilatory cycles at rest, without the need for patient effort.^17^

The main feature of volumetric capnography in an outpatient setting is the analysis of spontaneous breathing, which is very different from the forced maneuvers used in spirometry. During spontaneous breathing, natural variability in breathing patterns is observed, with cycle-to-cycle fluctuations in tidal volume, breathing times, and transpulmonary pressures.^18^ ^,^ ^19^ Although this variability represents an analytical challenge, it can provide additional diagnostic information on respiratory function.^20^

Volumetric capnography has proven cost-effective for monitoring patients with chronic respiratory diseases in outpatient settings. Because volumetric capnography is a noninvasive technique, its use can reduce the need for more complex tests and optimize resource allocation, serving as a complementary tool to spirometry in longitudinal patient monitoring.^21^

Most of the volumetric capnography devices that are currently available have been developed for use with mechanical ventilators in the ICU. There is a significant gap regarding portable devices for outpatient use. Therefore, the objective of the present study was to develop and validate a portable volumetric capnograph for collecting data on ventilatory mechanics in spontaneously breathing individuals in an outpatient setting. 

## METHODS

The study was conducted following all ethical principles set forth in Brazilian National Health Council Resolution No. 466/2012. The project was approved by the Research Ethics Committee of the *Universidade Estadual de Campinas*, located in the city of Campinas, Brazil (Protocol no. 6,897,044), and all volunteers gave written informed consent. 

### Hardware development

A portable volumetric capnograph was developed by integrating well-established, commercially available sensors for medical use. For respiratory flow measurement, the mainstream mode (all respiratory flow) was adopted with differential pressure measurement by variable orifice. The selected components were as follows:

a Hamilton^®^ pediatric/adult flow sensor (variable orifice; Hamilton Medical AG, Graubünden, Switzerland)
an SDP810-125PA differential pressure sensor (Sensirion AG, Stäfa, Switzerland) with an I2C digital output
a Capnostat 5 CO_2_ sensor (Philips Respironics, Murrysville, PA, USA) with an RS232 digital output
an Arduino UNO-R3^®^ microcontroller with an Atmega328P^®^ processor (Arduino, Monza, Italy)

**Figure 1:**
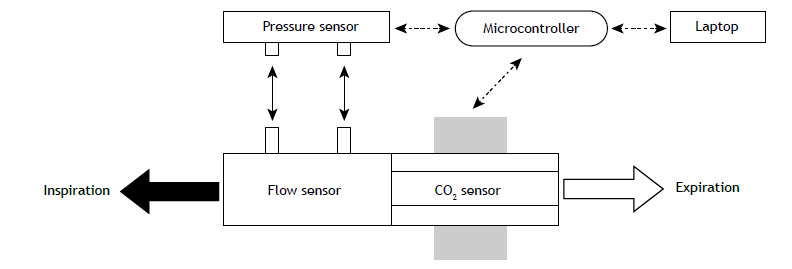
Illustration of the main components of our portable volumetric capnograph: a flow sensor connected to a differential pressure transducer, and a CO_2_ transducer connected to a microcontroller. The transducers have a digital output that integrates them with the microcontroller, which is connected to a laptop computer.

Figure 1 illustrates the main components used in developing the device. 

The Arduino UNO-R3^®^ is a microcontroller development board based on the ATmega328P chip, one of the most popular and widely used versions of the Arduino platform. It is especially used for prototyping; rapid development of automation concepts and sensor control (data reading and processing); and projects involving the internet of things. 

The differential pressure sensor was connected to the variable orifice flow meter via two flexible tubes, with an I2C digital communication protocol with the Arduino UNO-R3^®^ microcontroller. The CO_2_ sensor was connected to the same microcontroller via a 1.5 m cable using an RS232-TTL converter at 19200 baud. A USB port was used to connect the microcontroller to a laptop computer. 

### Firmware and software development

Firmware is a special type of low-level software that is permanently stored in hardware components-in the present study, the Arduino UNO-R3^®^ microcontroller-and provides basic instructions for the operation and control of electronic devices. 

In the present study, the set of instructions was written in the C++ programming language to manage communications, initialization, and data capture by the sensors. The sampling frequency was set at 100 Hz (10 ms), limited by the maximum rate available on the Capnostat 5. The flow sensor, with an internal resolution of 0.5 ms, had its mean values calculated for each 10 ms window. 

The user interface was developed in the Python programming language, with local storage (computer) of data in CSV files. Flow (L/min) and partial pressure of CO_2_ (PCO_2_, in mmHg) were recorded with synchronized timestamps of 10 ms. The volume of CO_2_ exhaled per cycle (VCO_2_/br) was calculated by equation 1, as follows:

$$VCO_{2}/br=\int_{II}^{FE}\frac{VT(t)\cdot PCO_{2}(t)dt}{Patm}$$ (1)

where *II* is the beginning of inspiration; *FE* is the end of expiration; VT is the tidal volume; Patm is the atmospheric pressure, the time differential used for integration being = 10 ms. 

### Static tests

Static tests were performed in the Ultrasound and Biomedical Instrumentation Laboratory of the Biomedical Engineering Center at the *Universidade Estadual de Campinas* under controlled temperature conditions (i.e., 24 ± 2°C). 

Inspiratory and expiratory flows were tested at 0, 5, 10, 15, 20, 25, 30, 35, 40, and 45 L/min, with 5 repetitions per point (totaling 100 measurements). Concentrations of CO_2_ were measured in steady state (constant CO_2_ concentration), with mixing performed from a cylinder. Medical air and CO_2_ flows were regulated with the aid of two needle valves and adjusted at each point using a Fluke VT650 gas analyzer (Fluke Biomedical, Everett, WA, USA) to adjust the flow and a CO_2_SMO Plus^®^ DX-8100 oxycapnograph (Philips Respironics) to adjust the CO_2_ concentration. The following CO_2_ concentrations were tested: 0, 5, 10, 20, 30, 40, 50, 60, 70, 80, and 100 mmHg, with constant flows of 5, 10, 15, 20, and 30 L/min; three measurements were taken at each point, totaling 165 measurements. 

### Tests with volunteers

Five healthy volunteers were recruited in accordance with the following inclusion criteria: being in the 18- to 40-year age bracket; having no respiratory diseases; being a nonsmoker. Exclusion criteria included pregnancy, use of respiratory medications, and recent respiratory infection. 

Each volunteer performed between 15 and 35 spontaneous breathing cycles in a sitting position, a total of 115 cycles being analyzed. The portable volumetric capnograph was connected in series with the reference equipment for simultaneous data capture. 

### Statistical analysis

The data were analyzed with Minitab software, version 19 (Minitab Inc., State College, PA, USA). For the static tests, the linear correlation coefficient (R^2^) was estimated for the confidence and prediction intervals, both with a 95% confidence level. 

Inspiration and expiration cycles were separated on the basis of the reversal of the respiratory flow in the table of raw data. Inspiration time (Ti) and expiration time (Te) were calculated by the time difference between the last and first measurements in each cycle. End-tidal CO_2_ (ETCO_2_) was obtained when the expiratory volume reached its maximum value (end of exhalation). Inspiration volume (Vi) and expired volume (Ve) were calculated by numerical integration of flow over time for each cycle, and VCO_2_/br was calculated by equation 1. 

For the tests with the five volunteers, the Mann-Whitney test was used to compare the means of respiratory parameters (ETCO_2_, Ti, Te, Vi, Ve, PEF, and VCO_2_/br). A value of p < 0.05 was considered significant. 

## RESULTS

**Figure 2:**
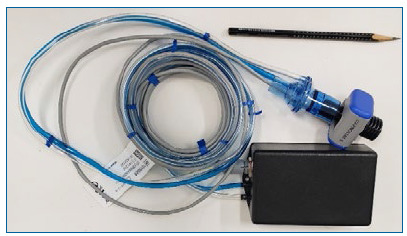
Photograph of our portable volumetric capnograph with sensors and connections.

Our portable volumetric capnograph was integrated into a compact box (15 × 10 × 5 cm) weighing 350 g. Total consumption was 1.4 W, powered via the computer USB port, with a startup time of 8 s. Figure 2 shows a photograph of the finished device. 

The characterization of the flow sensor demonstrated linear behavior with an R^2^ = 0.995 (95% CI, 0.993-0.997). A factor for correcting the slope of the line with a value of 1.438 was implemented in the firmware. 

The validation of the CO_2_ sensor showed an R^2^ = 0.995 (95% CI, 0.994-0.996), confirming equivalence with the reference equipment. 

**Figure 3:**
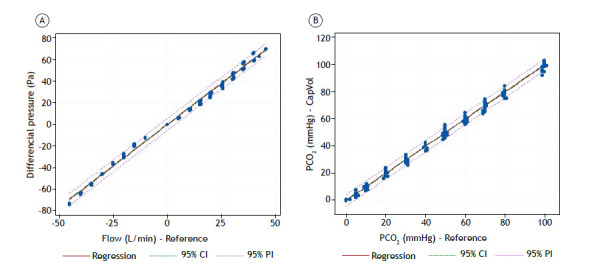
Graphs comparing our portable volumetric capnograph (CapVol) with a CO_2_SMO Plus^®^ DX-8100 oxycapnograph (Philips Respironics, Murrysville, PA, USA; reference equipment). In A, differential pressure (Pa) vs. flow for the reference equipment. In B, partial pressure of CO_2_ (PCO_2_) for CapVol vs. the reference equipment. PI: prediction interval.

Figures 3A and 3B illustrate the linear regression graphs of flow and CO_2_ concentration with the respective references. 

Table 1 shows the comparative results between our portable volumetric capnograph and the reference equipment, with no significant differences between the two (p > 0.05) in any of the parameters analyzed. 

**Table 1:** Results of a comparison between intracycle means of respiratory parameters of volunteers using a portable volumetric capnograph and a CO_2_SMO Plus® DX-8100 oxycapnograph (Philips Respironics, Murrysville, PA, USA).^a^

Variable	Volunteer				
	1 (n = 22)	2 (n = 34)	3 (n = 24)	4 (n = 18)	5 (n = 17)
ETCO_2_, mmHg					
CapVol	34.9 ± 0.8	32.1 ± 0.9	36.3 ± 1.2	36.5 ± 1.3	34.0 ± 1.0
CO_2_SMO	34.9 ± 0.8	32.3 ± 0.9	36.4 ± 1.4	35.8 ± 1.3	34.0 ± 1.2
p	0.37	0.11	0.82	0.13	0.68
Ti, s					
CapVol	1.8 ± 0.1	1.3 ± 0.2	2.5 ± 0.2	3.0 ± 0.5	2.3 ± 0.3
CO_2_SMO	1.8 ± 0.1	1.3 ± 0.2	2.5 ± 0.3	3.1 ± 0.6	2.3 ± 0.3
p	0.43	0.63	0.58	0.57	0.80
Te, s					
CapVol	2.4 ± 0.2	1.6 ± 0.1	3.1 ± 0.4	3.4 ± 0.6	3.3 ± 0.5
CO_2_SMO	2.4 ± 0.3	1.6 ± 0.1	3.1 ± 0.4	3.2 ± 0.5	3.4 ± 0.3
p	0.40	0.85	0.77	0.64	0.66
Vi, mL					
CapVol	844 ± 56	561 ± 76	1,041 ± 151	989 ± 289	986 ± 202
CO_2_SMO	824 ± 50	554 ± 75	1,032 ± 135	1,065 ± 233	1,046 ± 180
p	0.21	0.69	0.93	0.66	0.40
Ve, mL					
CapVol	787 ± 99	544 ± 59	1,009 ± 149	1,069 ± 237	857 ± 102
CO_2_SMO	798 ± 89	548 ± 53	1,053 ± 154	1,128 ± 242	920 ± 124
p	0.53	0.78	0.31	0.53	0.11
PEF, mmHg					
CapVol	32.5 ± 3.0	34.5 ± 3.2	28.9 ± 3.8	28.6 ± 4.4	23.0 ± 2.4
CO_2_SMO	31.5 ± 2.4	34.1 ± 2.5	30.1 ± 2.9	28.7 ± 3.8	24.0 ± 2.2
p	0.38	0.74	0.39	0.77	0.21
VCO_2_/br, mL					
CapVol	18.7 ± 3.6	11.2 ± 2.0	28.5 ± 6.3	33.2 ± 9.0	21.1 ± 3.9
CO_2_SMO	19.5 ± 3.3	11.8 ± 1.8	30.5 ± 6.2	33.8 ± 8.0	22.8 ± 4.5
p	0.67	0.08	0.26	0.82	0.27

n: number of respiratory cycles; ETCO_2_: end-tidal carbon dioxide; CapVol: portable volumetric capnograph; Ti: inspiration time; Te: expiration time; Vi: inspired volume per cycle; Ve: expired volume per cycle; VCO_2_/br: volume of CO_2_ expired per cycle. ^a^Data expressed as mean ± SD.

**Figure 4:**
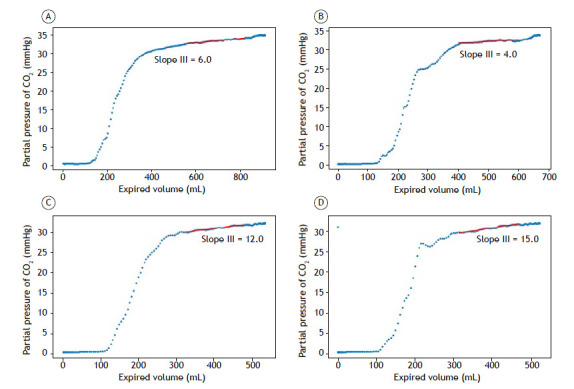
Capnography curves. In A, tidal volume vs. partial pressure of CO_2_ (PCO_2_), highlighting the position of slope III (volunteer 3, expiratory cycle 2). In B, tidal volume vs. PCO_2_, highlighting the position of slope III (volunteer 3, expiratory cycle 8). In C, tidal volume vs. PCO_2_, highlighting the position of slope III (volunteer 2, expiratory cycle 17). In D, tidal volume vs. PCO_2_, highlighting the position of slope III (volunteer 2, expiratory cycle 27).

Figures 4A, 4B, 4C, and 4D illustrate examples of capnography curves with the respective positions of slope III, calculated between 40% and 80% of tidal volume^22^ ^,^ ^23^ and showing the intercycle variability characteristic of spontaneous breathing. 

## DISCUSSION

The development of our portable volumetric capnograph represents an advance in the availability of volumetric capnography technology for data acquisition during spontaneous ventilation in an outpatient setting. The choice of well-established, commercially available components, integrated through a microcontroller platform, offers a good cost-benefit ratio. 

There is growing interest in the application of volumetric capnography for the diagnosis and monitoring of lung diseases,^11^ ^,^ ^14^ ^,^ ^16^ ^,^ ^24^ especially with the development of machine learning techniques.^7^ ^,^ ^9^ ^,^ ^25^

Zech et al.^26^ demonstrated that volumetric capnography can be reliably used in spontaneously breathing patients, showing a mean difference of −0.9 mmHg in the measurement of expired PCO_2_, with a correlation coefficient of 0.98 for reproducibility. The study supports the technical feasibility of the approach adopted in developing our portable volumetric capnograph, which achieved statistical equivalence with the reference equipment in all parameters evaluated. 

Kellerer et al.^17^ showed that volumetric capnography is a viable alternative when spirometry cannot be performed reliably. It allows the degree of functional impairment to be assessed in patients with obstructive pulmonary diseases, including COPD, asthma, and cystic fibrosis. This application is particularly relevant considering that our device does not require forced maneuvers by the patient, unlike spirometry, and its portability facilitates examination, benefiting severe lung disease patients who have difficulty moving and traveling to perform traditional ancillary tests, such as CT. 

Slope III indicator, calculated from the volumetric curve, has established clinical utility for the assessment of multiple lung diseases. Almeida et al.^27^ identified an increase in the slope of phase III normalized by tidal volume in asthma patients, suggesting ventilatory heterogeneity in the distal air spaces that may reflect chronic structural changes or acute reversible changes. Ribeiro et al.^28^ demonstrated the use of volumetric capnography for early detection of peripheral pulmonary obstruction in patients with cystic fibrosis, whereas Jarenbäck et al.^10^ validated its use for the diagnosis and grading of COPD. Moreira et al.^29^ ^,^ ^30^ described applications in the assessment of pulmonary thromboembolism, demonstrating the clinical versatility of indicators derived from volumetric capnography. 

The development of specific devices for spontaneous breathing is based on documented physiological differences between this modality and mechanical ventilation. Wolff et al.^31^ demonstrated that dead space ventilation is reduced during spontaneous breathing when compared with mechanical ventilation, this phenomenon being explained by a reduction in anatomical dead space and increased alveolar CO_2_ efficiency. 

Romero et al.^32^ showed that volumetric capnography can be used as an alternative to assess the severity of functional disorders in patients with COPD when spirometry cannot be performed reliably, reinforcing the need for specific devices for each ventilatory modality. 

Our technical validation showed a performance equivalent to that of the reference equipment, with an R^2^ = 0.995 for flow and CO_2_ measurements. These results are consistent with those reported for high-cost commercially available devices.^33^ ^,^ ^34^

The sampling rate of 100 Hz (10 ms) used in our portable volumetric capnograph exceeds the 50 Hz reported by Talker et al.^9^ for a device regulated in the European Union for COPD assessment. Higher sampling rates are especially better for slope II calculations, where the variation in PCO_2_ as a function of tidal volume is very pronounced. 

Sassmann et al.^35^ reported a resolution of 5 ms in high-fidelity equipment, suggesting that devices with higher time resolution provide more accurate analysis of expired CO_2_ kinetics. The best sampling rate for our device was limited by Capnostat 5, with no possibility of reducing the data acquisition time. 

The key finding of the present study was the variability of capnography curves during spontaneous breathing. Unlike controlled mechanical ventilation, where patterns are reproducible, spontaneous breathing has natural fluctuations that affect traditional physiological indicators. Although this variability represents a technical challenge for data processing, it contains valuable clinical information on individual breathing patterns and pulmonary heterogeneity. Tolnai et al.^36^ demonstrated that different respiratory rates in patients with spontaneous breathing produce significant variations in capnography indices, including changes in the slopes of phases II and III of the capnogram, corroborating the findings of the present study. 

Slope III, an important indicator for assessing obstructive diseases,^27^ ^,^ ^28^ showed intracycle variations of 25-50% and reflects the natural heterogeneity of spontaneous ventilation. Artificial intelligence algorithms can extract diagnostic information from this variability, as demonstrated in recent studies achieving an AUC of 0.93-0.99 for the classification of respiratory diseases.^37^

The prospects of our portable volumetric capnograph are promising in terms of development driven by miniaturization and portability, providing access to medical offices and clinics, with progressive cost reduction and democratization of access to these technologies. It establishes a technological basis for future studies focused on the following: clinical validation in patients with COPD, asthma, and other respiratory diseases; development of artificial intelligence algorithms for automated analysis of respiratory variability; and integration with telemedicine systems for remote monitoring. 

The incorporation of artificial intelligence in the automatic interpretation of capnograms is supported by recent studies. Feng et al.^25^ described growing applications of artificial intelligence and machine learning in chronic airway diseases, focusing on asthma and COPD. 

Zhou et al.^7^ demonstrated prediction of pulmonary function parameters based on combined algorithms, suggesting that the integration of volumetric capnography data with machine learning techniques can significantly amplify the clinical utility of ambulatory devices, democratizing the use of technology and significantly improving safety in monitoring patients during outpatient procedures. 

The design of a prototype, as proposed in the present study, represents only the first step in the development of an industrialized medical device. The transition to a commercially available product requires the implementation of a quality management system specific to medical devices,^38^ rigorous verification, validation, and biocompatibility processes.^39^

Because this was a preliminary study, it has limitations. One limitation is that the study included only five healthy volunteers, thus limiting its generalization to larger populations and those with respiratory diseases. Validation was performed in a controlled environment, and additional studies are needed in real outpatient conditions. In addition, no specific algorithms were developed for analyzing respiratory variability. 

In conclusion, our portable volumetric capnograph demonstrated technical capability for accurate flow and PCO_2_ measurements with a sampling rate of 100 Hz (10 ms), showing statistical equivalence with the commercially available reference equipment. The architecture based on commercially available components integrated by a microcontroller offers a cost-effective solution for ambulatory volumetric capnography. 

Although the variability observed in capnography curves during spontaneous breathing is challenging for traditional algorithms, it offers opportunities for the development of new respiratory indicators through artificial intelligence techniques. 

Future studies should focus on the development of adaptive algorithms for respiratory variability analysis and clinical validation in populations with respiratory diseases. 

## References

[1] Hashimoto N, Wakahara K, Sakamoto K (2021). The Importance of Appropriate Diagnosis in the Practical Management of Chronic Obstructive Pulmonary Disease. Diagnostics.

[2] Tan CL, Chan Y, Candasamy M, Chellian J, Madheswaran M, Sakthivel LP (2022). Unravelling the molecular mechanisms underlying chronic respiratory diseases for the development of novel therapeutics via in vitro experimental models. Eur J Pharmacol.

[3] Barnes PJ (2017). Cellular and molecular mechanisms of asthma and COPD. Clin Sci (Lond).

[4] CDC Chronic Obstructive Pulmonary Disease (COPD) Statistics.

[5] Qaseem A, Snow V, Shekelle P, Sherif K, Wilt TJ, Weinberger S (2007). Diagnosis and management of stable chronic obstructive pulmonary disease a clinical practice guideline from the American College of Physicians. Ann Intern Med.

[6] Global Initiative for Chronic Obstructive Lung Disease (GOLD) [homepage on the Internet] (2020). Global Strategy for Prevention, Diagnosis and Management of COPD: 2023 Report.

[7] Zhou R, Wang P, Li Y, Mou X, Zhao Z, Chen X (2022). Prediction of Pulmonary Function Parameters Based on a Combination Algorithm. Bioengineering.

[8] Exarchos KP, Beltsiou M, Votti CA, Kostikas K (2020). Artificial intelligence techniques in asthma a systematic review and critical appraisal of the existing literature. Eur Respir J.

[9] Talker L, Dogan C, Neville D, Lim RH, Broomfield H, Lambert G (2024). Diagnosis and Severity Assessment of COPD Using a Novel Fast-Response Capnometer and Interpretable Machine Learning. COPD.

[10] Jarenbäck L, Tufvesson E, Ankerst J, Bjermer L, Jonson B (2018). The Efficiency Index (EFFi), based on volumetric capnography, may allow for simple diagnosis and grading of COPD. Int J Chron Obstr Pulm Dis.

[11] Diniz OHG (2022). Volumetric Capnography History, Function and Clinical Uses. Open Access Library Journal.

[12] Howe TA, Jaalam K, Ahmad R, Sheng CK, Nik Ab Rahman NH (2011). The use of end-tidal capnography to monitor non-intubated patients presenting with acute exacerbation of asthma in the emergency department. J Emerg Med.

[13] Jaffe MB (2017). Using the features of the time and volumetric capnogram for classification and prediction. J Clin Monit Comput.

[14] Kremeier P, Böhm SH, Tusman G (2020). Clinical use of volumetric capnography in mechanically ventilated patients. J Clin Monit Comput.

[15] Talker L, Neville D, Wiffen L, Selim AB, Haines M, Carter JC (2023). Machine diagnosis of chronic obstructive pulmonary disease using a novel fast-response capnometer. Respir Res.

[16] Siobal MS, Ong H, Valdes J, Tang J (2013). Calculation of Physiologic Dead Space Comparison of Ventilator Volumetric Capnography to Measurements by Metabolic Analyzer and Volumetric CO2 Monitor. Respir Care.

[17] Kellerer C, Klütsch K, Husemann K, Sorichter S, Jörres RA, Schneider A (2020). Capnovolumetry in combination with clinical history for the diagnosis of asthma and COPD. Prim Care Respir Med.

[18] Veronez LF (2014). Capnografia volumétrica na avaliação de doenças crônicas pulmonares.

[19] Mauri T, Cambiaghi B, Spinelli E, Langer T, Grasselli G (2017). Spontaneous breathing a double-edged sword to handle with care. Ann Transl Med.

[20] Yoshida T, Amato MBP, Kavanagh BP (2018). Understanding spontaneous vs ventilator breaths: impact and monitoring. Intensive Care Med.

[21] Kodali BS (2014). Capnography Outside the Operating Room. Survey Anesthesiol.

[22] Tang Y, Turner MJ, Baker AB (2007). Systematic errors and susceptibility to noise of four methods for calculating anatomical dead space from the CO2 expirogram. Bri J Anaesth.

[23] Kars AH, Bogaard JM, Stijnen T, Vries de J, Verbraak AF, Hilvering C (1997). Dead space and slope indices from the expiratory carbon dioxide tension-volume curve. European Respir J.

[24] Verscheure S, Massion PB, Verschuren F, Damas P, Magder S (2016). Volumetric capnography lessons from the past and current clinical applications. Crit Care.

[25] Feng Y, Wang Y, Zeng C, Mao H (2021). Artificial Intelligence and Machine Learning in Chronic Airway Diseases Focus on Asthma and Chronic Obstructive Pulmonary Disease. Int J Med Sci.

[26] Verschuren F, Heinonen E, Clause D, Zech F, Reynaert MS, Liistro G (2005). Volumetric capnography reliability and reproducibility in spontaneously breathing patients. Clin Physiol Funct Imaging.

[27] Almeida CCB, Almeida-Júnior AA, Ribeiro MÂGO, Nolasco-Silva MT, Ribeiro JD (2011). Volumetric capnography to detect ventilation inhomogeneity in children and adolescents with controlled persistent asthma. J Pediatr (Rio J).

[28] Ribeiro MÂGO, Silva MTN, Ribeiro JD, Moreira MM, Almeida CCB, Almeida-Junior AA (2012). Volumetric capnography as a tool to detect early peripheric lung obstruction in cystic fibrosis patients. J Pediatr (Rio J).

[29] Moreira MM, Martins LC, Metze K, Pereira MV, Paschoal IA (2018). Near-fatal pulmonary embolism capnographic perspective. J Bras Pneumol.

[30] Moreira MM, Terzi RGG, Paschoal IA, Martins LC, Oliveira EP da L, Falcão ALE (2010). Thrombolysis in massive pulmonary embolism based on the volumetric capnography [Article in Portuguese] Arq Bras. Cardiol.

[31] Wolff G, Brunner J, Grädel E (1986). Gas exchange during mechanical ventilation and spontaneous breathing. Intermittent mandatory ventilation after open heart surgery. Chest.

[32] Romero PV, Rodriguez B, de Oliveira D, Blanch L, Manresa F (2007). Volumetric capnography and chronic obstructive pulmonary disease staging. Int J Chron Obstruct Pulmon Dis.

[33] Jaffe MB (2008). Infrared Measurement of Carbon Dioxide in the Human Breath "Breathe-Through" Devices from Tyndall to the Present Day. Anesth Analg.

[34] Schmalisch G (2016). Current methodological and technical limitations of time and volumetric capnography in newborns. Biomed Eng Online.

[35] Sassmann T, Kovacs G, Douschan P, Foris V, Gumpoldsberger M, John N Detection of structural pulmonary changes with real-time and high-fidelity analysis of expiratory CO2.

[36] Tolnai J, Rárosi F, Tóth I, Babik B, Novák Z, Peták F (2024). Relationships between capnogram parameters by mainstream and sidestream techniques at different breathing frequencies. Sci Rep.

[37] McDowell A, Kang J, Yang J, Jung J, Oh YM, Kym SM (2022). Machine-learning algorithms for asthma, COPD, and lung cancer risk assessment using circulating microbial extracellular vesicle data and their application to assess dietary effects. Exp Mol Med.

[38] Kramer DB, Xu S, Kesselheim AS (2012). How Does Medical Device Regulation Perform in the United States and the European Union A Systematic Review. PLOS Med.

[39] Williams DF (2008). On the mechanisms of biocompatibility. Biomaterials.

